# Cerebrolysin Ameloriates Cognitive Deficits in Type III Diabetic Rats

**DOI:** 10.1371/journal.pone.0064847

**Published:** 2013-06-19

**Authors:** Gehan S. Georgy, Noha N. Nassar, Hanaa A. Mansour, Dalaal M. Abdallah

**Affiliations:** 1 Department of Pharmacology, National Organization of Drug Control and Research (NODCAR), Giza, Egypt; 2 Department of Pharmacology and Toxicology, Faculty of Pharmacy, Cairo University, Cairo, Egypt; Centre national de la recherche scientifique, France

## Abstract

Cerebrolysin (CBL), a mixture of several active peptide fragments and neurotrophic factors including brain-derived neurotrophic factor (BDNF), is currently used in the management of cognitive alterations in patients with dementia. Since Cognitive decline as well as increased dementia are strongly associated with diabetes and previous studies addressed the protective effect of BDNF in metabolic syndrome and type 2 diabetes; hence this work aimed to evaluate the potential neuroprotective effect of CBL in modulating the complications of hyperglycaemia experimentally induced by streptozotocin (STZ) on the rat brain hippocampus. To this end, male adult Sprague Dawley rats were divided into (i) vehicle- (ii) CBL- and (iii) STZ diabetic-control as well as (iv) STZ+CBL groups. Diabetes was confirmed by hyperglycemia and elevated glycated haemoglobin (HbA1c%), which were associated by weight loss, elevated tumor necrosis factor (TNF)-α and decreased insulin growth factor (IGF)-1β in the serum. Uncontrolled hyperglycemia caused learning and memory impairments that corroborated degenerative changes, neuronal loss and expression of caspase (Casp)-3 in the hippocampal area of STZ-diabetic rats. Behavioral deficits were associated by decreased hippocampal glutamate (GLU), glycine, serotonin (5-HT) and dopamine. Moreover, diabetic rats showed an increase in hippocampal nitric oxide and thiobarbituric acid reactive substances versus decreased non-protein sulfhydryls. Though CBL did not affect STZ-induced hyperglycemia, it partly improved body weight as well as HbA1c%. Such effects were associated by enhancement in both learning and memory as well as apparent normal cellularity in CA1and CA3 areas and reduced Casp-3 expression. CBL improved serum TNF-α and IGF-1β, GLU and 5-HT as well as hampering oxidative biomarkers. In conclusion, CBL possesses neuroprotection against diabetes-associated cerebral neurodegeneration and cognitive decline via anti-inflammatory, antioxidant and antiapototic effects.

## Introduction

The decline in cognitive function associated with Alzheimer's disease is viewed as “Type 3 Diabetes” that is linked to decreased insulin production [1]. The hippocampus, a key area for learning and memory is considered a special target for alterations associated with diabetes [2]. This area is most vulnerable to uncontrolled peripheral hyperglycemia manifest as decreased general cognitive performance, memory impairment and atrophy [3]. Persistent hyperglycemia supports the production of advanced glycation end products (AGEs) that is a pivotal player in diabetic complications [4]. Both the process of glycation as well as AGEs via binding to its membrane receptors generate proxidant and pro-inflammatory statuses [5], hence mediating neuronal degeneration [6]. Evidence exist that neuronal loss and the allied neurotransmitter alterations are core events underlying both impaired cognitive and neuropsychiatric symptoms [7].

Cerebrolysin (CBL), a mixture of several active peptide fragments and neurotrophic factors, is currently used in the management of several central disorders [8] as well cognitive alterations in patients with dementia [9]. The drug improves cognitive performance in Alzheimer's patients, an effect that may be linked to either halting autoimmune activation and/or enhanced neurogenesis [10], [11]. On one hand, CBL reduces T-lymphocyte-induced apoptosis [12]. while, on the other hand, it acts similar to endogenous neurotrophic factors [13], promoting synaptic and neuronal plasticity as well as cytoskeletal stability [14]. Notably, CBL comprises brain-derived neurotrophic factor (BDNF) in its composition [15]; the latter plays a pivotal in the survival, maintenance and neuronal cell differentiation [16]. Furthermore, this neurotrophic factor that maintains long-term potentiation (LTP) and neurogenesis is abundantly expressed in the hippocampus [17]. Collectively, CBL enhances neurogenesis in the hippocampal subgranular zone of the dentate gyrus by augmenting survival of neural progenitor cells [11], [18] and improves learning and memory [19].

The current investigation focused on the decline in cognitive functions associated with streptozotocin (STZ) induced diabetes. The present study aimed thus to investigate the potential protective effect of CBL on cognitive deficits and the associated possible underlying pathways linked to diabetic complications evoked by acute administration of STZ. To this end, insulin growth factor (IGF)-1β, oxidative stress, inflammation and apoptotic biomarkers, as well as hippocampal neurotransmitters were targeted. In addition, histopathological changes induced by STZ and the possible correction by CBL were assessed.

## Materials and Methods

### Animals

Adult male Sprague Dawley rats (200–240 g) were used in the present study. Animals were obtained from the breeding colony at the animal facility of the National Organization for Drug Control and Research (NODCAR, Giza, Egypt). Rats were kept under controlled environment, at a constant temperature (23±2°C), humidity (60±10%) and light/dark (12/12 hr) cycle. They were allowed standard rat chow and tap water ad libitum. Animal handling and experimental protocols were approved by the Research Ethical Committee of the Faculty of Pharmacy, Cairo University (Cairo, Egypt) and comply with the Guide for the Care and Use of Laboratory Animals [20].

### Induction of Experimental Diabetes and Treatment

Animals were divided into 4 groups (n = 20–22, each group), which were further subdivided into 3 subsets (n = 6–8) for (i) neurotransmitters, (ii) oxidative stress, and inflammatory parameters estimation as well as (iii) histopathological and immunohistochemical examinations. Body weight, blood glucose, glycated hemoglobin (HbA1c%) and behavioral evaluations were carried out in all animals (n = 20–22, each group).The four groups were divided as follows: (i) vehicle-control (equivalent volume of the citrate buffer i.p. in place of STZ and (ii) CBL-control (2.5 ml/kg, i.p.) for 4 weeks [21]. Rats in the two remaining groups were rendered diabetic by a single intraperitoneal injection of 40 mg/kg STZ in freshly prepared 0.1 M citrate buffer (pH 4.5) after an overnight fast, then given 5% glucose solution for 24 hr to prevent initial hypoglycaemic mortality [22]. Three days after STZ injection, diabetes was confirmed using a digital glucometer (Bionime Rightest GM100; Taiwan) on blood droplets obtained from the tail vein. Animals showing fasting blood glucose higher than 210 mg/dl were used in the current study [22]. Following the initial 4 weeks of diabetes induction by STZ, rats were allocated as (iii) STZ diabetic-control, receiving no treatment [23] and (iv) STZ+CBL for 4 additional weeks.

### Behavioral Tests

**Morris Water Maze.** Memory retention was performed using Morris water maze paradigm [24] five days prior to sacrifice. For four consecutive days, each rat was daily trained twice and the escape latency was recorded during 120 sec period; those which failed to find the platform within the examination period, an escape latency of 120 sec was taken. On the 5^th^ day, the platform was removed and each rat was placed in the maze to record the swimming time during 120 sec in platform quadrant.
**Passive Avoidance Test.** Passive avoidance test was carried out as previously described [25]. Briefly, during the training sessions, three min adaptation and aversion trials in the apparatus were achieved. Twenty four and 48 hr, following the training sessions, the avoidance of the dark shock-associated compartment and the latency to enter the dark compartment were recorded to present as memory retention trial within 3 min. Nonetheless, a latency of 3 min was given to those animals that did not enter the dark compartment within the experimentation period.

### Determination of Body Weight Change, Blood Glucose and Glycated Haemoglobin (HbA1c%)

Body weight was monitored weekly in all animals under study. Twenty-four hrs following the last treatment, sera were separated for glucose determination according to Trinder [26]. Whole blood was lysed and hemoglobin was retained on a cation exchange resin for HbA1c% determination [27] using the reagent kit provided by Biosystems (Barcelona, Spain). HbA1c was eluted using sodium azide in phosphate buffer (pH 6.5) and quantified at 415 nm.

### Determination of Serum Tumor Necrosis Factor (TNF)-α and Insulin Growth Factor (IGF)-1β

Serum TNF-α and IGF-1β (Boster Biological Technology, Wuhan, China) were measured using commercially available rat enzyme-linked immunosorbent assay (ELISA) kits according to manufacturers' instruction.

### Histopathological, and Caspase (Casp)-3 Immunohistochemical Examinations

Brains were collected from representative animals in each group, and immediately fixed in 10% phosphate-buffered formaldehyde. Subsequently, brains were embedded in paraffin; 5 µm sections were prepared, stained with haematoxylin and eosin (H&E), and examined microscopically. In the hippocampal areas, the number of cells was counted in a fixed field size (60 000 µm^2^ for CA1/hilus and 10 000 µm^2^ for CA3) [28]. Other sections were examined for Casp-3 expression in the hippocampus. Antigen retrieval was performed by boiling tissue sections in 10 Mm citrate buffer (pH 6; 20 min) followed by cooling at room temperature (RT). Sections were then incubated with rabbit polyclonal anti-Casp-3 (CPP32) antibody (1∶200; Thermo Fisher Scientific, Labvision, Fremont, USA) for 30 min at RT followed by an additional 1 h to a biotinylated mouse secondary antibody. Amplification of the bound secondary antibody was performed using a Vector Elite ABC kit (Vector Laboratories, Burlingame, CA, USA). Next, 0.02% DAB was used to visualize the antibody-biotin-avidin-peroxidase complexes. Sections were then mounted onto gelatin-coated slides and air dried overnight at RT, cover-slipped and mounted using Permount [29]. The numbers of Casp-3-positive cells were then expressed as cells per mm^2^ of the cross sectional area of the hippocampal area.

### Tissue Sampling

At the end of experiment, 24 hr following the last treatment, rats were sacrificed and both hippocampi were isolated and stored at −80°C. For the determination of neurotransmitters, a 10% (w/v) homogenate was prepared in a 75% methanol for HPLC. Each homogenate was centrifuged at 1000×g (4°C) for 10 min. The resultant supernatant was divided into two halves; the first was dried using vacuum (70 millipore) at RT and its residues were derivatized for the determination of brain amino acids, whereas the second half was used for monoamines determination. In another subset, hippocampi were homogenized in 10% (w/v) phosphate buffer (pH 7.6) for the assay of the other biochemical parameters.

### Determination of Hippocampal Neurotransmitters

Hippocampal amino acids aspartate (ASP), glutamate (GLU), and glycine (GLY) were detected by HPLC, according to Heinrikson and Meredith [30] using the precolumn isothiocyanate derivatization technique. Briefly, brain amino acids were estimated using a fully automated high-pressure liquid chromatography system (HPLC; Perkin- Elmer, MA, USA). Reconstituted brain residues (2∶2∶1 mixture (v) of methanol: 1 M sodium acetate: triethylamine) were redried under vacuum. One aliquot was subject to a 20 min derivatization performed using a 7∶1∶1∶1 mixture (v) of methanol: triethylamine: double-distilled deionized water: phenylisothiocyanate, redried under vacuum, then reconstituted with sample diluents [5∶95 mixture (v) of acetonitrile: 5 mM phosphate buffer (pH = 7.2). Sonicated and filtered (0.45 µm; Millipore) samples were run on a Pico-Tag physiological free amino acid analysis C18 (300 mm×3.9 mm i.d.) column from Waters (MA, USA) and a binary gradient of Eluents 1 and 2 (Waters) were used. Column temperature (46±1°C) and a constant flow rate (1 ml/min) were sustained throughout the experiment. A sample volume of 20 µl was injected, and the corresponding peak was detected at 254 nm. Another aliquot was used for the estimation of monoamines namely serotonin (5-HT), dopamine (DA) as well as norepinephrine (NE) according to Kontur et al. [31]. The supernatant was filtered (0.45 µm; Millipore), and then 20 µl were injected into an ODS-reversed phase column (C18, 25×0.46 cm; i.d. 5 µm). The mobile phase consisted of potassium phosphate buffer: methanol [97∶3 (v)] at a flow rate of 1.5 ml/min, and the corresponding peaks were detected at 270 nm.

### Determination of Hippocampal Thiobarbituric Acid Derivatives (TBARS), Non Protein Thiols (NPSH), and Nitric Oxide (NO)

Hippocampal TBARS level was measured as described by Deniz et al. [32]. Briefly, thiobarbituric acid in trichloroacetic was added to the homogenate and boiled for 30 min, cooled and centrifuged at 1000×g. The absorbance of the supernatant was determined at 535 nm. NPSH content was determined according to the procedure of Prins and Loose [33]. Homogenates were deproteinated, centrifuged and NPSH in the supernatant was reacted with Ellman's reagent that was measured at 412 nm. Hippocampal NO was determined using the reagents of kit provided by Biodiagnostic (Cairo, Egypt). Sample protein was precipitated using ethanol for 48 hr at 4°C. To the clear supernatant, vanadium trichloride was added to reduce nitrate to nitrite, followed by the addition of Griess reagent; and the absorbance was measured at 540 nm.

### Statistical analysis

Results were expressed as mean ± S.E.M (n = 6–22). Statistical analysis was performed using SPSS, version 13®. Statistical analysis was carried out using one-way analysis of variance (ANOVA) followed by Tukey–Kramer Multiple Comparison Test, while two-way ANOVA followed by Bonferroni Post Hoc Test was used to analyze the body weight as well as Morris water maze escape latency. The results were considered significant at *P*<0.05.

## Results

### Effect of CBL on Weekly Body Weight, HbA1c%, as well as Serum Glucose, IGF-1β, and TNF-α in Diabetic Rats

**Figure 1:**
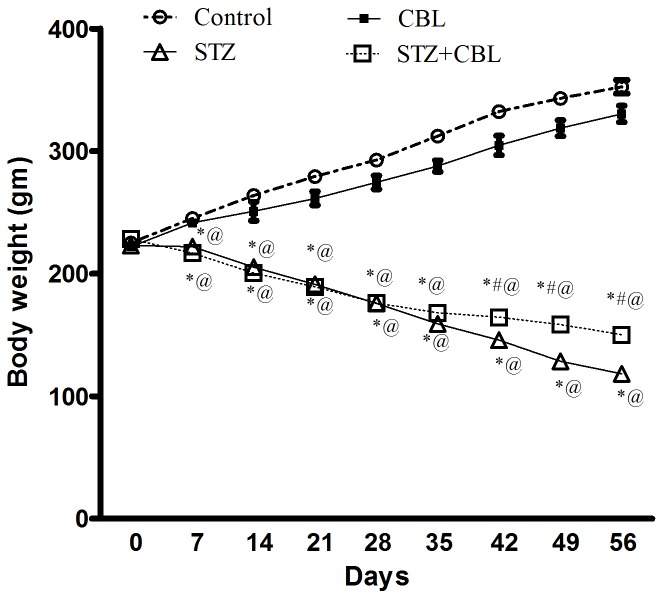
Effect of 4 weeks daily administration of cerebrolysin (CBL; 2.5 ml/kg) on body weight in control and STZ diabetic rats. Data represents mean (n = 20–22) ± S.E.M. ^*#@^ *P*<0.05 compared to the vehicle- (CONT), STZ-,and CBL-control groups, respectively. Statistical analysis was carried out by two way ANOVA followed by Bonferroni Post Hoc Test.

At the end of the experiment, rats given STZ, showed marked reduction in body weight by 67% (118.2 g±1.11; *P*<0.0001) compared to vehicle control (352.5 g±5.61; Fig. 1). Such an effect was in part halted by CBL administration to STZ-treated rats mounting to 27% (150 g±1.2; *P*<0.0001) from diabetic animals. Hyperglycemia was induced by STZ administration (2fold increase, 315.6 mg/dl±20.1; *P*<0.0001), an effect that was associated by elevation of HbA1c% to 73% (7.61±0.4; *P*<0.0001) compared to their respective vehicle controls (Table 1). Despite that CBL administration to STZ-treated rats partially suppressed HbA1c% by 22% (5.9±0.3; *P*<0.0001), it failed to affect the hyperglycemia evoked by STZ (Table 1). Serum IGF-1β was reduced by 65% in diabetic rats (2541.7 pg/ml±104.4; *P*<0.0001) where CBL administration to diabetic animals partially improved its level, an effect reaching 53% (3900.3 pg/ml±254.8; *P* = 0.01) from STZ-treated rats (Table 1). On the other hand, serum TNF-α was increased by 75% (364.2 pg/ml±29.4; *P*<0.0001) in STZ-treated rats, an event that was almost restored by CBL administration to diabetic animals (232.5 pg/ml±14.8; *P* = 0.001; Table 1).

**Table 1:** Effect of 4 weeks daily administration of cerebrolysin (CBL; 2.5 ml/kg) on serum glucose, glycated haemoglobin (HbA1c%), as well as serum TNF-α and IGF-1β in STZ diabetic rats.

Serum TNF-α (pg/ml; n = 6–8)	Serum IGF-1β (pg/ml; n = 6–8)	HbA1c% (n = 20–22)	Serum Glucose (mg/dl; n = 20–22)	Groups
208.33±15.4	7191.67±376.5	4.39±0.3	104.12±3.7	CONT
170.83±31.2	6983.33±345.1	3.76±0.2	83.90±4.5	CBL
364.17±29.4*@	2541.67±104.4*@	7.61±0.4*@	315.59±20.1*@	STZ
232.50±14.8#	3900.33±254.8*#@	5.92±0.3*#@	300.85±10.6*@	STZ+CBL

Data represents mean ± S.E.M.

*#@ *P*<0.05 compared to the vehicle- (CONT), STZ-, and CBL-control groups, respectively.

Statistical analysis was carried out by one way ANOVA followed by Tukey- Kramer Multiple Comparison Test.

### Effect of CBL on STZ-induced Behavioral Alterations in Diabetic Rats

**Figure 2:**
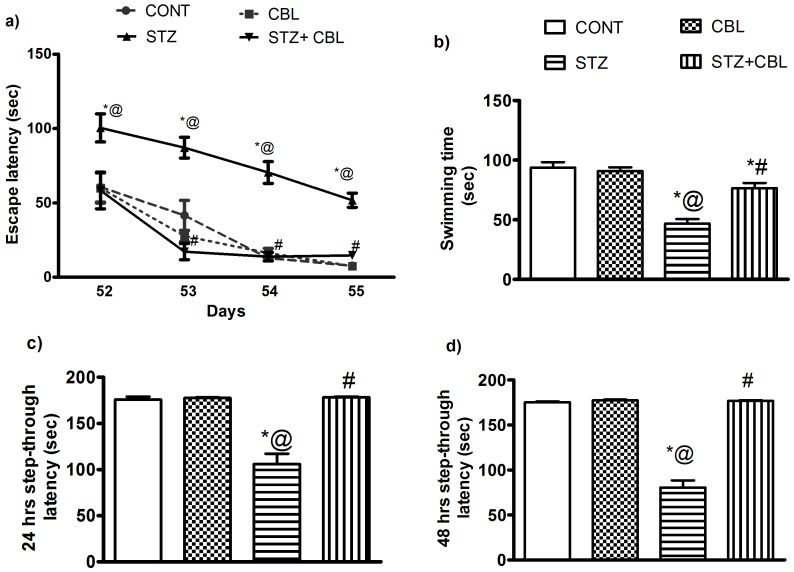
Effect of 4 weeks daily administration of cerebrolysin (CBL; 2.5 ml/kg) on escape latency (a) and swimming time (b) in Morris water maze test in control and STZ diabetic rats (c) 24 hr and (d) 48 hr step through latency in the passive avoidance test Data represents mean (n = 20–22) ± S.E.M. ^*#@^ *P*<0.05 compared to the vehicle- (CONT), STZ-,and CBL-control groups, respectively. Statistical analysis of escape latency was carried out by two way ANOVA followed by Bonferroni Post Hoc Test. Statistical analysis of swimming time probe test was carried out by one way ANOVA followed by Tukey- Kramer Multiple Comparison Test.

During the Morris water maze training course, the escape latency was increased in diabetic rats (Fig. 2a), while in the probe trial, STZ-treated rats spent significantly less time in the target quadrant (46.8 sec±3.8; *P*<0.0001) than animals in other groups (Fig. 2b). In the training course, on the second, third and fourth days, diabetic rats receiving CBL showed improvement in reaching the platform, effects which reached normal values (Fig. 2a). In the probe test, CBL significantly partially reduced diabetes-induced memory deficits by 63% (76.5 sec±4.3; *P*<0.001) compared to STZ-treated animals (Fig. 2b). In the passive avoidance test, STZ suppressed the 24 (Fig. 2c) and 48 hr (Fig. 2d) entrance latency to the dark compartment by 40 and 54% (106 sec±11.1and 80.4 sec±7.9 respectively; *P*<0.0001) after training. Administration of CBL in diabetic rats enhanced retention latency in the test sessions 24 and 48 hr reaching 68 and 120% (178.3 sec±0.8 and 176.7 sec±0.7, respectively; *P*<0.0001), compared with STZ-diabetic rats (Fig. 2c–d).

### Effect of CBL on STZ-induced Histological Alterations and Casp-3 Immunoreactivity

**Figure 3:**
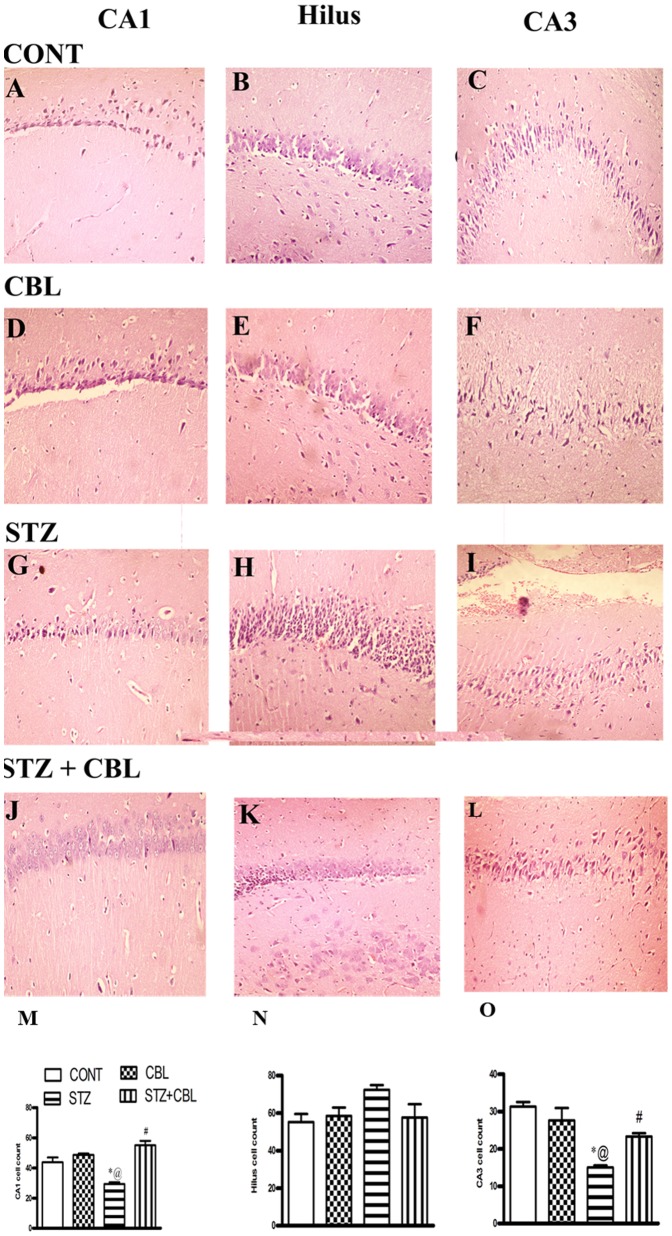
Effect of 4 weeks daily administration of cerebrolysin (CBL; 2.5 ml/kg) on histological appearance (a–l) and neuronal cell count (m–o) in CA1, hilus, and CA3 hippocampal areas in control and STZ diabetic rats. Data represents mean (n = 6) ± S.E.M. Cells were counted in a fixed field size 60 000 µm^2^ for CA1/hilus and 10 000 µm^2^ for CA3.^*#@^ *P*<0.05 compared to vehicle- (CONT), STZ-and CBL-control groups,respectively. Statistical analysis was carried out by one way ANOVA followed by Tukey- Kramer Multiple Comparison Test.

**Figure 4:**
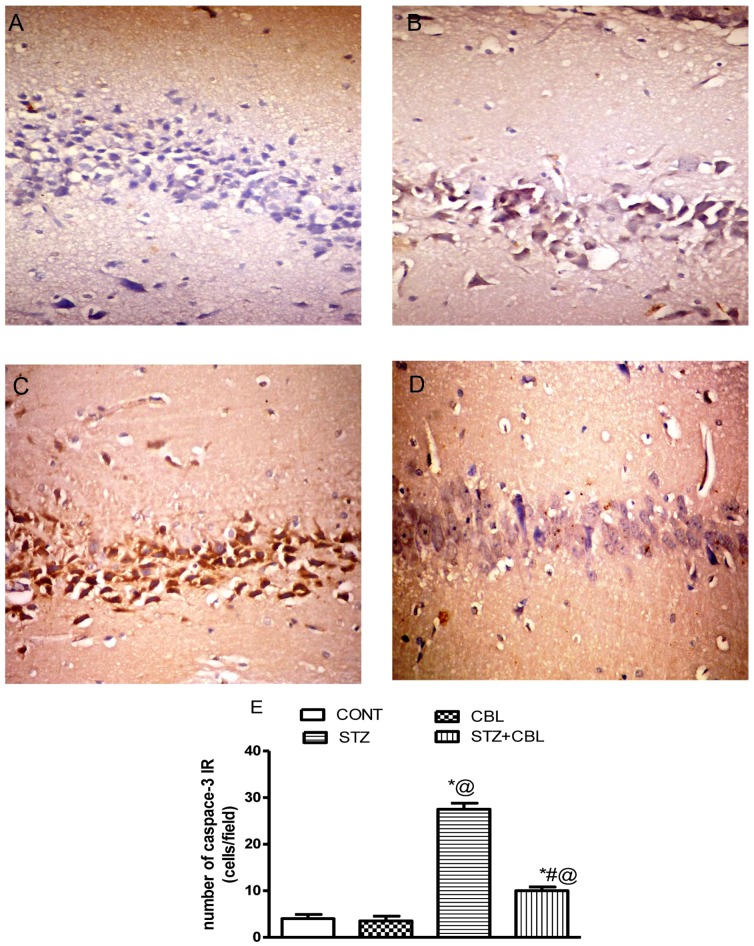
Photomicrographs depicting immunohitochemical staining for Casp-3 expression in (a) vehicle-, (b) CBL-, (c) STZ-control and (d) STZ+CBL hippocampal area. Animals receiving STZ showed positive Casp-3 expression, which was markedly reduced by CBL treatment. Panel (e) represents mean (n = 6) ± S.E.M of hippocampal Casp-3 cell count. Cells were counted in a fixed field size 10 000 µm^2^. ^*#@^ *P*<0.05 compared to vehicle (CONT)-, STZ-and CBL-control groups, respectively. Statistical analysis was carried out by one way ANOVA followed by Tukey- Kramer Multiple Comparison Test.

Both vehicle control (Fig. 3a–c) as well as CBL (Fig. 3d–f) treated rats showed normal histological appearance and distribution of neuronal cells in CA1, hilus, and CA3 hippocampal areas (Fig. 3m–o). Degenerative changes were shown in all three hippocampal areas (CA1, hilus, and CA3) of STZ-diabetic rats (Fig. 3g–i). The CA1 region displayed few pyknotic cells with reduced neuronal cell count (*P* = 0.018; Fig. 3g and m). Moreover, the hilus revealed more pyknotic cells as well as mild congested capillaries (Fig. 3h) with less prominent neuronal loss versus the CA1 area (Fig. 4m and n). Notably, the CA3 area bared marked reduction of pyramidal cells (*P* = 0.0012) corroborating intracellular edema and obvious capillary congestion (Fig. 3i and o). Treatment with CBL in STZ-diabetic rats showed apparently normal cellularity in CA1 area, fewer pyknotic cells in hilus (Fig. 3j and k). Furthermore, the CA3 area showed some improvement in the appearance of the pyramidal cells as well as retained cellular count, compared to STZ-treated rats (Fig. 3l and o).

Furthermore, the hippocampal area of rats receiving STZ showed Casp-3 immunoreactive positive cells mounting to nearly six folds compared to control (*P*<0.0001; Fig. 4a–c and e). Treatment with CBL resulted in a marked reduction of positive Casp-3 cells in the hippocampus of diabetic rats (*P*<0.0001; Fig. 4d).

### Effect of CBL on Hippocampal Neurotransmitters and Oxidative Stress Biomarkers in Diabetic Rats

**Figure 5:**
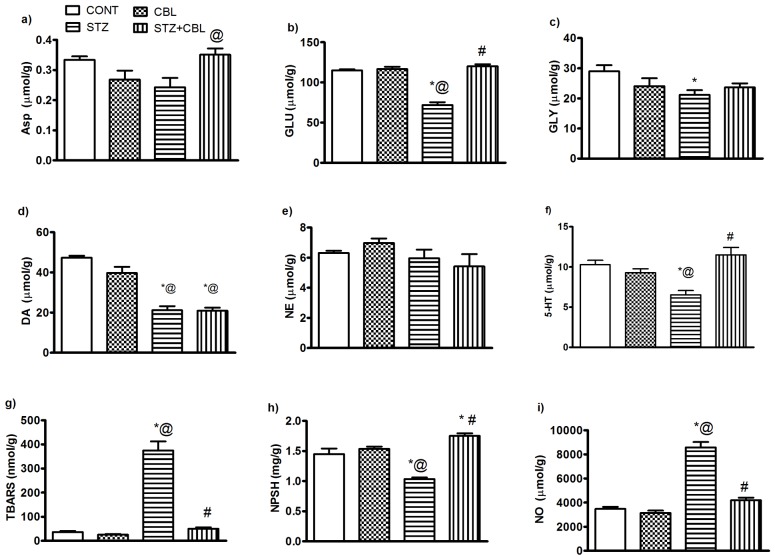
Effect of 4 weeks daily administration of cerebrolysin (CBL; 2.5 ml/kg) on hippocampal aminoacids content ASP(a), GLU(b), GLY(c), DA (d), NE (e), 5-HT (f), TBARS (g), NPSH (h) and NO (i) in control and STZ diabetic rats. Data represents mean (n = 6–8) ± S.E.M. ^*#@^ *P*<0.05 compared to vehicle- (CONT), STZ-and CBL-control groups, respectively. Statistical analysis was carried out by one way ANOVA followed by Tukey- Kramer Multiple Comparison Test.

STZ animals showed no change in either ASP or NE hippocampal contents (Figs. 5a, e), however, hyperglycemia reduced the concentrations of GLU 43% (71.9 µmol/g±3.5; *P*<0.0001;Fig. 5b), GLY 27% (21.2 µmol/g±2.05; *P* = 0.046; Fig. 5c), DA 55% (21.2 µmol/g±2.05; *P*<0.0001; Fig. 5d) as well as 5-HT 37% (6.5 µmol/g±0.54; *P* = 0.003; Fig. 5f) compared to their respective vehicle control rats.CBL administration enhanced ASP, GLU and 5-HT contents to different extents in STZ-diabetic to vehicle control counterparts (Figs. 5a, b and f).

Diabetes produced marked elevation in hippocampal TBRAS (Fig. 5g) and NO (Fig. 5i) levels by ten and 2.5 fold (374.7 nmol/g±37.6 and 8581 µmol/g±440.8, respectively; *P*<0.0001) versus control animals. Both biomarkers were almost restored by CBL administration to STZ- rats. Nonetheless, NPSH (Fig. 5h) was reduced in diabetic rats by 29% (1.03 mg/g±0.03; *P*<0.0001) an effect that was increased after CBL administration by 69% (1.75 mg/g±0.05; *P*<0.0001) compared to STZ animals (Fig. 5h).

## Discussion

To the best of the authors' knowledge, this is the first report demonstrating enhanced cognitive memory functions by CBL administration in an STZ-induced hyperglycemic model. The central findings of the current study reveal that CBL halts STZ behavioral induced deficits despite its inability to revert the associated hyperglycemic action; however, it partially prevented the elevation in HbA1c%. The STZ induced behavioral impairments correlate with Casp-3 expression as well as histopathological changes in the hippocampus showing cellular death and neurodegeneration; alterations which were held in check by CBL. Moreover, the treatment partially raised IGF-1β and normalized TNF-α serum levels when given to diabetic rats. Enhanced learning and memory by CBL in diabetic rats paralleled elevations in hippocampal excitatory amino acids to different extents as well as 5-HT.

Diabetes was confirmed by hyperglycemia and elevation of HbA1c%, a long-term diabetic control index, 4 weeks post STZ administration to rats. These findings are in line with another report [34] and were associated by retardation in animal growth observed as reduction in body weight in the present study. Notably, in the current investigation, STZ administration increased serum TNF-α that inhibits the uptake of circulating free fatty acids and accelerates lipolysis in adipose tissue [35], thus lending a plausible explanation for the reduction in body weight; both former events were impeded by CBL administration in the present work. The anti-inflammatory effect of CBL is supported by the work of Alvarez et al. [36], who reported decreased TNF-α level in Alzheimer's patients.

Uncontrolled diabetes/hyperglycemia as shown by elevated blood glucose as well as increased HbA1c%, in the present work, correlate significantly with decline in cognitive function [4], [37]–[38]. Excess glucose induces the major HbA1c [39] that releases iron, thus generating free radicals and other AEGs evoking both reactive oxygen and nitrogen species formation [40]. Iron overload and excessive free radical production increase the blood brain barrier (BBB) permeability [41]. In diabetes, AGEs elicit oxidative stress through up-regulation and interaction with receptor for AGEs (RAGE) via sustained activation of transcriptional factor nuclear factor (NF)-κb [42]. The latter further induces expression of inducible nitric oxide synthase (iNOS) and TNF-α [43] thus providing explanation for the observed increase in NO and TNF-α. The proinflammatory cytokine, when produced systemically, may gain access to the hippocampus via the disrupted BBB [44]. In a vicious cycle, TNF-α has been shown to induce iNOS expression, which in conjunction with reactive oxygen species exacerbates tissue injury [45]. A plausible mechanism for the observed Casp-3 expression and neurodegenerative changes may stem from the excessive free radical generation and TNF- α [46]. Apart from free radicals produced by AGEs [47], the auto-oxidation of elevated intracellular glucose levels [48] as well as DA turn over [49] add to the oxidant pool manifest as increased TBARS as well as NO in face of a decline in NPSH in this work. An escalated DA turnover that contributes to enhanced oxidative stress burden [49] is displayed in the current study by the decrease in DA without a change in NE level in the STZ animals.

Worthy of note, the hippocampus bears the greatest abundance for BDNF, which plays a pivotal role in hippocampal neurogenesis and consequently cognitive functions [50]. BDNF, a component of CBL [15], has been shown to reduce HbA1c when administered intermittently in obese diabetic mice [51]. Notably, in the present work, CBL reduced HbA1c, via its antioxidant power reflected by a reduction in lipid peroxidation and NO as well as hindering NPSH depletion in the hippocampus, that parallel improvement in cognition and memory. Circulating IGF-1β gains access to the brain via its transporters regulating, thus hippocampal IGF-1β levels [52]. In the present study, STZ reduced circulating IGF-1β production, an effect that has been partially reversed by CBL administration. The latter event is suggestive of enhanced pyruvate formation, a metabolite of the glycolytic pathway [53]. Pyruvate reduces oxidative stress by scavenging H_2_O_2_, which in turn is reflected as reduced depletion of NPSH, in addition to a decline in TBARS [54]. Another merit to CBL is its ability to reduce TNF-α, an event shown in this study, and one pivotal factor for central neuronal death [55]. Directly, CBL is reported to reduce TNF-α [36] and indirectly via reduction of BBB permeability [56] thus limiting entry of TNF-α into the brain, which further contributes to halting excess NO production and tissue injury reflected as behavioral improvement in the current work.

In addition to the changes in behavior associated with altered glucose metabolism, clinical and experimental studies implicate neurotransmitters alterations in the adverse effects on cognitive functions [57], [58]. Notably, a positive correlation between DA depletion and decline in cognitive functions exists. This view is supported by findings linking the degeneration of dopaminergic neurons in Parkinson's disease to decreased hippocampal neurogenesis that precedes movement restrictions [59]; and, hence, cognitive impairment in learning and memory tests [58]. Consequently, the observed alterations in behavior as well as neurotransmitters, in this study, corroborated neuronal degeneration in the hippocampus as manifest by Casp-3 expression and histopathological findings. Conversely, in the current study, CBL by conserving DA and decreasing Casp-3 expression produced neuroprotection, reflected as amelioration of the associated behavioral deficits. The latter effect is in line with previous findings from aged apolipoprotein E-deficient mice [60] and transgenic Alzheimer's animal model receiving CBL [61].

Noteworthy, deficit in serotonergic [62] as well as glutamatergic [63] neurotransmission decrease hippocampal neurogenesis, which is pivotal for cognition. Accordingly, this might lend a plausible explanation for the decline in behavioral functions seen in this investigation; with the waning in 5-HT as well as GLU content in STZ exposed rats. In the present investigation, the utilized neuropeptide, CBL, via enhancing GLU/5-HT concentrations contributing, further, to improved behavior. It is well established that LTP and memory consolidation are closely related to both glutametergic as well as serotonergic transmission [64]–[65]. Notably, a shortfall in LTP consequent to deficits in hippocampal synaptic transmission correlates with decreased expression of n-methyl-D-aspartate (NMDAR) subunits NR2A and NR2B [66]. Though a subtle reduction in ASP was only observed in STZ-treated animals, the CBL diabetic rats showed its enhanced levels, an effect promoting better glutaminergic neurotransmission [67] and hence behavioral amendment. However, one might argue that decreased acetylcholine (ACh) is the major player in incidence and progression of cognitive decline [68]. Interestingly, findings from the current study reveal that CBL did not correct the reduction in ACh induced by STZ (data not shown). These findings are supported by the study of Comim et al. [69] showing that hippocampal BDNF, the CBL neurotrophin component [15], may contribute to memory impairment without alterations in ACh.

Take all together, the current investigation implicates a pivotal role for CBL against cognitive decline associated with “Type 3 Diabetes”. This afforded protection corroborates preservation of neuronal cells via halting Casp-3 expression and ameliorating oxidative stress damage, inflammation, as well as abnormal neurotransmission.
